# Cerebrospinal fluid biomarkers that reflect clinical symptoms in idiopathic normal pressure hydrocephalus patients

**DOI:** 10.1186/s12987-022-00309-z

**Published:** 2022-02-05

**Authors:** Heikki Lukkarinen, Anna Jeppsson, Carsten Wikkelsö, Kaj Blennow, Henrik Zetterberg, Radu Constantinescu, Anne M. Remes, Sanna-Kaisa Herukka, Mikko Hiltunen, Tuomas Rauramaa, Katarina Nägga, Ville Leinonen, Mats Tullberg

**Affiliations:** 1 Department of Neurosurgery, Institute of Clinical Medicine – Neurosurgery, Kuopio University Hospital and University of Eastern Finland, Kuopio, Finland; 2 Hydrocephalus Research Unit, Department of Clinical Neuroscience, Institute of Neuroscience and Physiology, The Sahlgrenska Academy, University of Gothenburg, Gothenburg, Sweden; 3 Department of Clinical Neuroscience, Institute of Neuroscience and Physiology, The Sahlgrenska Academy, University of Gothenburg, Gothenburg, Sweden; 4 Department of Psychiatry and Neurochemistry, Institute of Neuroscience and Physiology, The Sahlgrenska Academy, University of Gothenburg, Gothenburg, Sweden; 5 Clinical Neurochemistry Laboratory, Sahlgrenska University Hospital, Mölndal, Sweden; 6 Department of Neurodegenerative Disease, UCL Institute of Neurology, London, UK; 7 UK Dementia Research Institute at UCL, London, UK; 8 Hong Kong Center for Neurodegenerative Diseases, Hong Kong, China; 9 Unit of Clinical Neuroscience, Neurology, University of Oulu, Oulu, Finland; 10 Medical Research Center, Oulu University Hospital, Oulu, Finland; 11 Department of Neurology, Kuopio University Hospital and University of Eastern Finland, Kuopio, Finland; 12 Institute of Biomedicine, University of Eastern Finland, Kuopio, Finland; 13 Department of Pathology, Kuopio University Hospital and University of Eastern Finland, Kuopio, Finland; 14 Department of Acute Internal Medicine and Geriatrics, Linköping University, Linköping, Sweden

**Keywords:** iNPH, Idiopathic normal pressure hydrocephalus, Biomarkers

## Abstract

**Background:**

The relationship between cerebrospinal fluid (CSF) biomarkers and the clinical features of idiopathic normal pressure hydrocephalus (iNPH) has been inconclusive. We aimed to evaluate CSF biomarkers reflecting Alzheimer’s disease (AD)-related amyloid β (Aβ) aggregation, tau pathology, neuroinflammation and axonal degeneration in relation to the clinical features of pre- and post-shunt surgery in iNPH patients.

**Methods:**

Mini Mental State Examination (MMSE) scores and gait velocity were evaluated pre- and postoperatively in cohorts of 65 Finnish (FIN) and 82 Swedish (SWE) iNPH patients. Lumbar CSF samples were obtained prior to shunt surgery and analysed for soluble amyloid precursor protein alpha (sAPPα) and beta (sAPPβ); amyloid-β isoforms of 42, 40 and 38 (Aβ_42_, Aβ_40_, Aβ_38_); total tau (T-tau); phosphorylated tau (P-tau_181_); neurofilament light (NfL) and monocyte chemoattractant protein 1 (MCP1).

**Results:**

Preoperative patient characteristics showed no significant differences between patients in the FIN and SWE cohorts. Patients in both cohorts had significantly improved gait velocity after shunt surgery (*p* < 0.0001). Low CSF T-tau and absence of apolipoprotein E ε4 predicted over 20% gait improvement postoperatively (*p* = 0.043 and *p* = 0.008). Preoperative CSF T-tau, P-tau_181_ and NfL correlated negatively with MMSE scores both pre- (*p* < 0.01) and post-surgery (*p* < 0.01). Furthermore, T-tau, NfL and Aβ_42_ correlated with MMSE outcomes (*p* < 0.05). Low preoperative CSF P-tau_181_ (*p* = 0.001) and T-tau with NfL (*p* < 0.001 and *p* = 0.049) best predicted pre- and postoperative MMSE scores greater than or equal to 26.

**Conclusions:**

CSF biomarkers of neurodegeneration appeared to correlate with pre- and postoperative cognition, providing a window into neuropathological processes. In addition, preoperative CSF neurodegeneration biomarkers may have potential in the prediction of gait and cognitive outcomes after shunt surgery.

## Background

Idiopathic normal pressure hydrocephalus (iNPH) is characterized by a symptom triad of gait dysfunction, dementia and incontinence, accompanied by enlarged ventricles [1, 2]. Symptom progression can be reversed with CSF shunt surgery [3, 4]. The common symptom triad and shunt-surgery outcomes are usually quantified by symptom domain classifying grading scales. Regardless of careful patient selection, not all patients have favourable surgical outcomes [5, 6]. It has been suggested that poor shunt surgery outcomes are derived from commonly coexisting neurodegenerative diseases, such as Alzheimer’s disease (AD) or vascular degeneration [7, 8].

There are several CSF biomarkers assessed for diagnostic and predictive purposes in the field of neurodegenerative diseases. For iNPH patients, it has been shown that the lumbar CSF composition of low tau and APP-derived proteins together with high MCP1 can distinguish iNPH patients from cognitively intact individuals and patients with other neurodegenerative diseases [9]. However, the association of a wider repertoire of AD biomarkers with postsurgery clinical features in iNPH are still somewhat inconclusive [10–13].

In the current literature, postoperative gait velocity has been found to be associated with lumbar CSF T-tau and P-tau_181_ collected preoperatively [11, 13]. Regarding cognitive decline, studies have shown the predictive value of P-tau_181_, the Aβ_42_/P-tau_181_ ratio and the Aβ_38_/Aβ_42_ ratio [12, 13], with a higher P-tau_181_ correlating with a poorer cognitive outcome. In another study [14], CSF Aβ_42_ was shown to correlate with postoperative MMSE values.

The role of the axonal degeneration biomarker neurofilament-light (NfL) as a symptom predictor in iNPH patients has not been studied widely either. In AD patients, higher plasma NfL levels have been shown to be associated with poorer Mini Mental State Examination (MMSE) scores [15], and plasma Nfl and CSF NfL levels are the best single predictors of cognition in AD patients [16]. On the other hand, a recent study found no significant correlation between neuropsychiatric symptoms and CSF NfL in AD [17]. For vascular dementia, however, there was a negative correlation between CSF NfL and neuropsychiatric performance [17].

Since there are tendencies for biomarkers to enhance the accuracy of diagnosis and prediction of outcome in iNPH, establishing a more precise, specific preoperative biomarker combination able to predict a favourable shunt surgery outcome would be highly beneficial.

### Objective

In this study, we aimed to evaluate preoperatively-obtained CSF biomarkers reflecting AD-related Aβ aggregation, tau pathology, neuroinflammation and axonal degeneration and their associations with pre- and postoperative clinical features in Finnish and Swedish iNPH cohorts.

## Methods

### Study populations

**Fig. 1:**
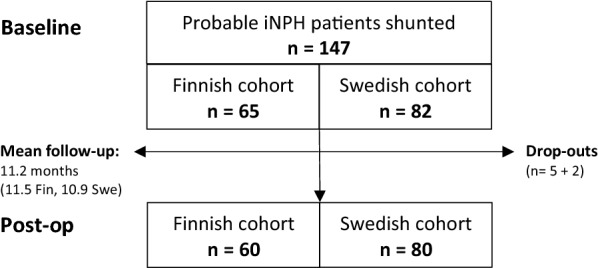
Flow chart. Flow chart presenting the cohorts and participants. Baseline represents the presurgery examination and Postoperative the visits after surgery. INPH: idiopathic normal pressure hydrocephalus; n: number; post-op: postoperative; pre-op: preoperative

In all, 65 and 82 consecutive shunted patients with probable iNPH diagnosed by the Relkin criteria [18] at Kuopio University Hospital and the Hydrocephalus Research Unit, Sahlgrenska University Hospital in Gothenburg were included (Fig. 1). The requirement for ventriculoperitoneal CSF shunt surgery was assessed using previously described protocols [9, 19]. The clinical features of cohorts were evaluated pre- and postoperatively using Mini Mental State Examination (MMSE) and gait velocity (m/s) along with the iNPH grading scale (NPHGS, 0–12) [20] for Finnish patients and the iNPH scale (NPHS, 0–100) [21] for Swedish patients. A favourable shunt surgery outcome was defined as a decrease of ≥ 1 point in iNPHGS and an increase of > 5 points in iNPH scale. Comorbidities of type 2 diabetes mellitus (DM2), hypertension and cardiac diseases were also registered preoperatively. The diagnostic protocols and CSF sampling were executed 3 months prior to shunt surgery. The postsurgery evaluation was performed 3 and 12 months postoperatively in the Finnish cohort where the 3-month value was used if the 12-month visit was missing. In the Swedish cohort, postsurgery evaluation was performed approximately 6 months postoperatively. The mean follow-up times were 11.5 months for the Finnish cohort and 10.9 months for the Swedish cohort. During the follow-up, 7 participants withdrew or died (5 from the Finnish cohort and 2 from the Swedish cohort). In addition, venous blood samples were obtained for *APOE* genotyping, and DNA was isolated using a commercial kit according to the manufacturer’s protocol (Illustra Blood GenomicPrep Mini Spin Kit, GE Healthcare, Little Chalfont, UK). The extracted samples were analysed by the standard PCR method [22].

### Biomarker analysis

Preoperative CSF samples were obtained by lumbar puncture in both cohorts and retained in 10 ml polypropylene tubes. Samples were centrifuged, aliquoted and frozen in a temperature-controlled − 80 °C freezer. There was no blood contamination seen in the samples obtained.

The CSF biomarker measurements were performed at the Clinical Neurochemistry Laboratory at Sahlgrenska University Hospital, Mölndal, Sweden. The laboratory technicians were board-certified and blinded to the clinical data. All experiments were executed on the same plate in one round of experiments and using the same batch of reagents. Commercial kits were used to perform the biomarker concentration analysis. CSF T-tau and P-tau_181_ were measured by INNOTEST ELISAs using the kit manufacturer’s protocol (Fujirebio, Ghent, Belgium). For Aβ_38_, Aβ_40_, Aβ_42_, sAPPα, sAPPβ and MCP1 analysis, electrochemiluminescence (ECL) assays were used, as described by the manufacturer [23] (Meso Scale Discovery Rockville, MD, USA). The NfL concentration was measured with an in-house ELISA, as previously described [24].

### Statistics

The group comparisons performed for mean values and frequencies were performed by t tests for continuous variables and x^2^ or Wilcoxon’s signed ranks test for discrete variables. Pearson correlation coefficients (r) were assessed for linear dependency analyses. Since CSF concentrations of T-tau, P-tau_181_, NfL, Aβ_38_, Aβ_40_, Aβ_42_, sAPPα, sAPPβ and MCP1 were concordant in both cohorts, we pooled the cohorts for correlation analysis. For pre- and postoperative comparisons, we used analysis of covariance (ANCOVA) with the confounding covariates of age, sex and *APOE* genotype. Receiver operating characteristics (ROC curves) and binary logistic regression were used to model MMSE performance and gait outcome. Pre- and postoperative MMSE with gait outcome were dichotomized by cut-offs of 26 by the mild dementia value commonly used and 20% improvement in gait. ROC analysis of biomarkers, dichotomized MMSE and gait was performed. We aimed to identify optimal biomarker cut-offs by estimating Youden’s indices and favouring balanced sensitivity and specificity. Univariate modelling was performed, and significant variables with age, sex and *APOE* genotype were used in the multiple regression model. Multicollinearity diagnostics were used for variables included in the multiple regression model. All tests calculated were two-sided and considered significant with a *p value* less than 0.05. The statistical analyses were performed in IBM SPSS Statistics 27.00 for IOS.

## Results

**Fig. 2:**
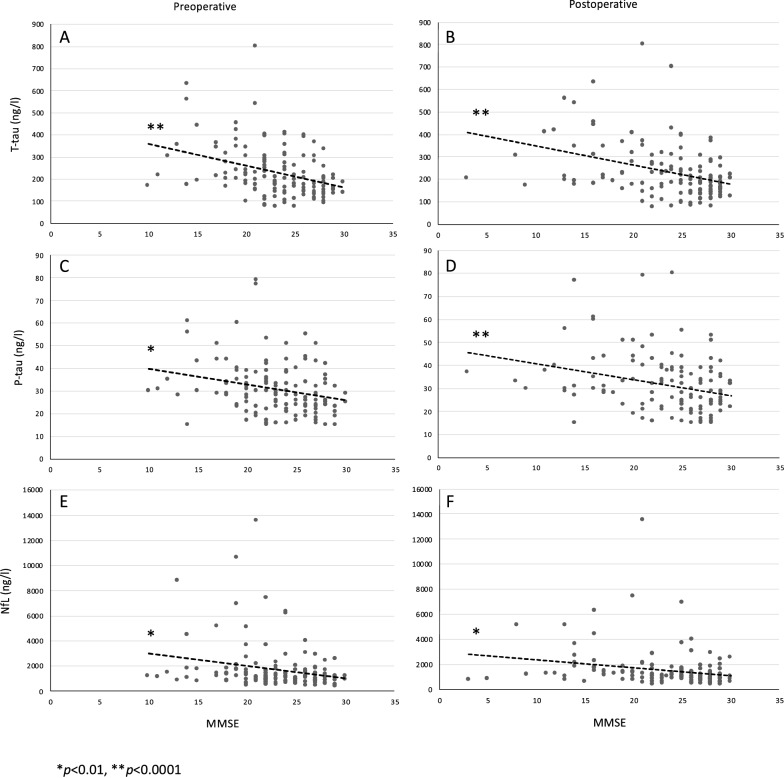
Pre- and postoperative correlation of Mini Mental State Examination and biomarkers. Graphs of concentration of lumbar CSF biomarkers of T-tau (A, B), P-tau_181_ (C, D) and NfL (E, F): preoperative—(**A**, **C**, **E**) and postoperative (**B**, **D**, **F**) MMSE values. Significant results are highlighted with asterisk (*p < 0.01, **p < 0.0001). Linear trendlines are drawn to visualize the correlations. T-tau: total tau protein; P-tau: phosphorylated at threonine 181 tau protein; NfL: neurofilament-light; MMSE: mini mental state examination

**Fig. 3:**
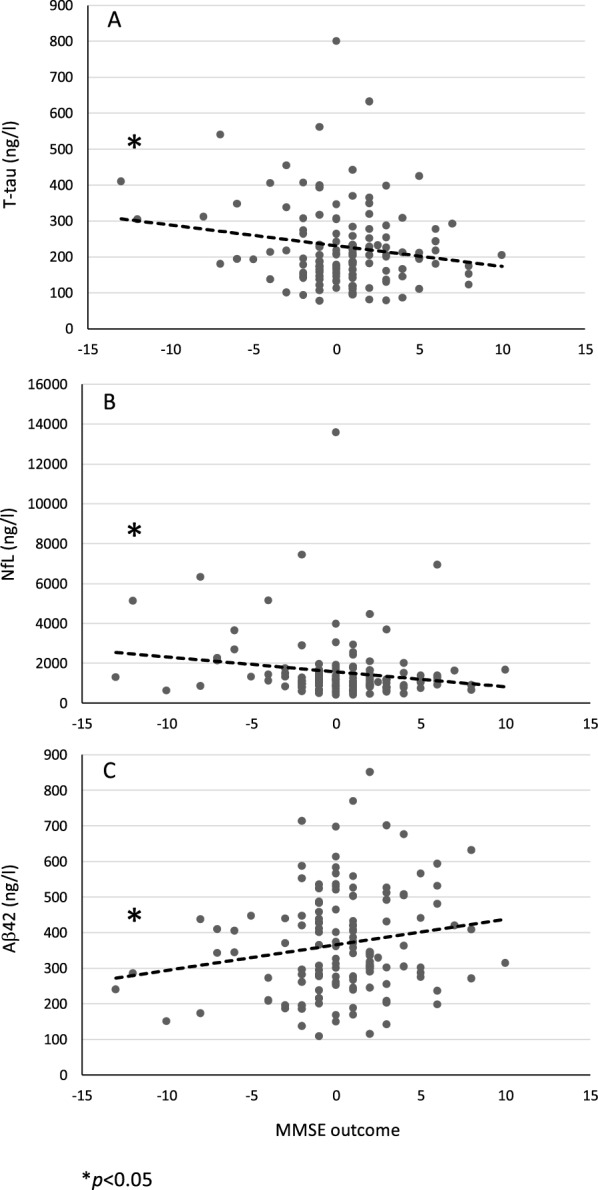
Correlation of Mini Mental State Examination outcome and biomarkers. Graphs of preoperatively obtained lumbar CSF biomarkers of T-tau (**A**), NfL (**B**) and Aβ_42_ (**C**) correlated with MMSE outcomes (T-tau r = − 0.18, NfL r = − 0.17, Aβ_42_ r = 0.18). Negative outcome is presented as a decrease in X-axis. Significant results are highlighted with asterisk (*p < 0.05). Linear trendlines are drawn to visualize the correlations. T-tau: total tau protein; NfL: neurofilament-light; Aβ42: Amyloid-β 42; MMSE: mini mental state examination

**Fig. 4:**
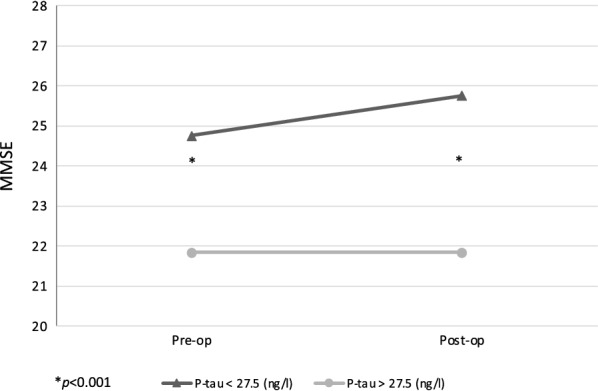
Pre-and postoperative Mini Mental State Examination results grouped by P-tau_181_ concentrations. Longitudinal change of pooled MMSE results with CSF P-tau_181_ derived grouping presented in chart. Cutoff used for grouping was 27.5 ng/l. Low P-tau_181_ group presented as triangles and high P-tau_181_ group as circles. Significant results are presented by asterisk (*p < 0.001). MMSE, mini mental state examination; Pre-op: preoperative; Post-op: postoperative; P-tau: phosphorylated at threonine 181 tau protein

The patient demographic and clinical characteristics, *APOE* genotype and mean CSF biomarker values are presented in Table 1. No significant differences were seen in demographic or clinical characteristics or *APOE* genotype between the Finnish and Swedish cohorts. In addition, CSF concentrations of T-tau, P-tau_181_, NfL, Aβ_38_, Aβ_40_, Aβ_42_, sAPPα, sAPPβ and MCP1 were concordant in both cohorts. Correlation analysis was assessed for pooled cohorts (Fig. 2, Table 2). Preoperative MMSE results correlated negatively with T-tau (r = − 0.36, p < 0.0001, Fig. 2A), P-tau_181_ (r = − 0.26, p = 0.002, Fig. 2C) and NfL (r = − 0.23, p = 0.006, Fig. 2E). Comparable results were seen between the postoperative MMSE values and biomarkers of T-tau (r = − 0.37, p < 0.0001, Fig. 2B), P-tau_181_ (r = − 0.30, p < 0.0001, Fig. 2D) and NfL (r = − 0.23, p = 0.006, Fig. 2F). The postsurgery MMSE outcome correlated negatively with T-tau (r = − 0.18, p = 0.046, Fig. 3A) and NfL (r = − 0.17, p = 0.045, Fig. 3B) and positively with Aβ_42_ (r = 0.18, p = 0.38, Fig. 3C). We compared MMSE results between P-tau_181_ cut-off 27.5 ng/l derived groups and a 4.0% increase (mean score 24.8–25.8) was seen in low P-tau_181_ group during the follow-up whereas the high P-tau_181_ group remained similar (mean score 21.8) (Fig. 4). Both pre- and postsurgery MMSE values differed between patients in the P-tau_181_-derived groups (Pre, p < 0.001; Post, p < 0.001). In addition, the P-tau_181_ groups showed divergent Aβ_42_ concentrations of 321 ng/l in the low P-tau_181_ group and 415 ng/l in the high P-tau_181_ group (p < 0.001). *The APOE* genotype correlated only with Aβ_42_ (r = − 0.27, p = 0.003, Table 2).

**Table 1:** Patient characteristics

Patient characteristics	Finnish cohort	Swedish cohort	
	n = 65	n = 82	*p*-value
Age (y); mean (SD)	72.4 (7.4)	73.0 (7.2)	0.60
Male sex; n (%)	37 (57)	53 (65)	0.34
Weight (kg); mean (SD)	79.2 (15.4)	79.2 (19.8)	0.99
Height (cm); mean (SD)	168.4 (7.8)	169.1 (18.8)	0.79
Diabetes mellitus T2; n (%)	24 (38)	19 (24)	0.099
Hypertonia; n (%)	41 (63)	44 (54)	0.29
Cardiac disease; n (%)	21 (32)	26 (32)	0.98
MMSE; mean (SD)			
Pre-op	22.6 (4.2)	23.6 (4.4)	0.16
Post-op	22.8 (5.4)	23.9 (5.7)	0.22
Gait velocity (m/s); mean (SD)			
Pre-op	0.60 (0.26)	0.66 (0.27)	0.26
Post-op	0.80 (0.31)	0.84 (0.36)	0.51
*APOE*- genotype; n (%)			0.32
ε2/ε2	0 (0)	1 (1.9)	
ε2/ε3	7 (10.9)	5 (9.3)	
ε2/ε4	3 (4.7)	4 (7.4)	
ε3/ε3	42 (65.6)	26 (48.1)	
ε3/ε4	10 (15.6)	16 (29.6)	
ε4/ε4	2 (3.1)	2 (3.7)	
NPHGS improvement; n (%)	34 (52)		
NPHS improvement; n (%)		48 (59)	

Biomarkers			
T-tau (ng/l); mean (SD)	222.0 (111.4)	244.9 (130.5)	0.28
P-tau (ng/l); mean (SD)	30.5 (12.2)	31.9 (12.4)	0.50
NfL (ng/l); mean (SD)	1573.8 (1661.5)	1717.3 (1962.8)	0.64
Aβ38 (ng/l); mean (SD)	1471.4 (515.0)	1526.0 (518.9)	0.53
Aβ40 (ng/l); mean (SD)	3697.5 (1161.4)	3799.8 (1193.5)	0.60
Aβ42 (ng/l); mean (SD)	367.4 (151.4)	364.3 (137.8)	0.90
sAPPα (ng/l); mean (SD)	416.6 (212.7)	446.2 (177.6)	0.37
sAPPβ (ng/l); mean (SD)	305.2 (142.2)	321.0 (121.1)	0.47
MCP1 (ng/l); mean (SD)	463.2 (122.1)	492.2 (109.4)	0.13

Patient characteristics of Finnish and Swedish cohorts presented as mean and standard deviation (SD) or as number and percent. *P values* are analyzed between the cohorts using t test for continuous variables and chi-square for categorical variables. T2: Diabetes mellitus type 2; MMSE: mini mental state examination; Pre-op: preoperative; Post-op: postoperative; APOE: apolipoprotein E; NPHGS: normal pressure hydrocephalus grading scale; NPHS: normal pressure hydrocephalus scale; T-tau: total tau protein; P-tau: phosphorylated at threonine 181 tau protein; NfL: neurofilament-light; Aβ38: Amyloid-β 38; Aβ40: Amyloid-β 40; Aβ42: Amyloid-β 42; sAPPα: soluble amyloid precursor protein α; sAPPβ: soluble amyloid precursor protein β, MCP1: monocyte chemoattractant protein 1

**Table 2:** Correlation coefficients of CSF biomarkers of T-tau, P-tau_181_, NfL, Aβ-isoforms (38, 40, 42), sAPPα, sAPPβ and MCP1 *among* with the *APOE*-genotype, age, MMSE and gait velocity

Pearson correlation coefficients (r)		P-tau	NfL	Aβ38	Aβ40	Aβ42	sAPPα	sAPPβ	MCP1	*APOE*	Age	MMSE Pre-op	MMSE Post-op	MMSE Outcome	Gait Pre-op
	T-tau														
T-tau															
P-tau	.904^**^														
NfL	.501^**^	.300^**^													
Aβ38	.624^**^	.708^**^	.157												
Aβ40	.622^**^	.720^**^	.156	.964^**^											
Aβ42	.291^**^	.365^**^	.128	.771^**^	.784^**^										
sAPPα	.414^**^	.505^**^	.114	.638^**^	.645^**^	.481^**^									
sAPPβ	.412^**^	.511^**^	.053	.647^**^	.639^**^	.442^**^	.926^**^								
MCP1	.355^**^	.324^**^	.249^**^	.296^**^	.288^**^	.229^**^	− .024	.021							
*APOE*	− .058	− .008	− .042	− .051	− .050	− .268^**^	− .001	.004	− .144						
Age	.320^**^	.268^**^	.125	.317^**^	.370^**^	.194^*^	.194^*^	.181^*^	.087	.036					
MMSE Pre-op	− .364^**^	− .263^**^	− .231^**^	− .092	− .127	− .016	.011	.021	− .025	− .018	− .185^*^				
MMSE Post-op	− .372^**^	− .300^**^	− .228^**^	− .038	− .067	.107	.036	.079	.030	− .049	− .069	.731^**^			
MMSE Outcome	− .177^*^	− .141	− .173^*^	.090	.086	.179^*^	.120	.147	.048	− .076	− .013	− .114	.595^**^		
Gait Pre-op	− .155	− .143	− .251^**^	− .128	− .182^*^	− .194^*^	− .077	.001	− .012	.159	− .314^**^	.430^**^	.246^**^	− .057	
Gait Post-op	− .325^**^	− .249^**^	− .253^**^	− .137	− .189^*^	− .096	.025	.084	− .076	− .068	− .354^**^	.529^**^	.491^**^	.143	.787^**^

Significant results are highlighted by asterisk (*p < 0.05, **p < 0.01). *APOE*-correlation is analyzed by scaling the allele combinations by the risk (22 = 1, 23 = 2, 33 = 3, 24 = 4, 34 = 5, 44 = 6). CSF: cerebrospinal fluid; MMSE: mini mental state examination; Gait: gait velocity in m/s; Pre-op: preoperative; Post-op: postoperative; APOE: apolipoprotein E; T-tau: total tau protein; P-tau: phosphorylated at threonine 181 tau protein; NfL: neurofilament-light; Aβ38: Amyloid-β 38; Aβ40: Amyloid-β 40; Aβ42: Amyloid-β 42; sAPPα: soluble amyloid precursor protein α; sAPPβ: soluble amyloid precursor protein β, MCP1: monocyte chemoattractant protein 1

**Fig. 5:**
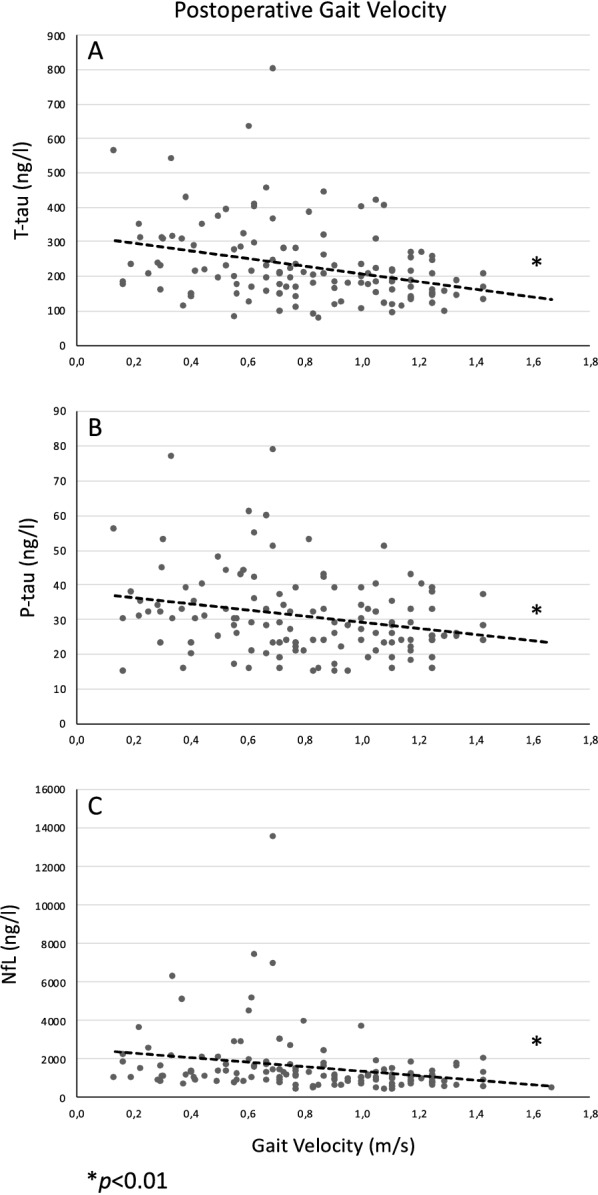
Chart of postoperative correlation of biomarkers with gait velocity. Preoperatively obtained lumbar CSF biomarkers of T-tau (**A**), P-tau_181_ (**B**) and NfL (**C**) correlated negatively (T-tau r = − 0.33, P-tau r = − 0.25, NfL r = − 0.25) with postoperative gait velocity (m/s) and presented in XY-chart. Significant correlations are highlighted with asterisk (*p < 0.01). Linear trendlines are drawn to visualize the correlations. T-tau: total tau protein; P-tau: phosphorylated at threonine 181 tau protein; NfL: neurofilament-light

In gait velocity, both cohorts improved significantly after surgery (by 38% FIN, 33% SWE) (p < 0.0001) (Table 1). In the pooled cohort, only NfL showed a negative correlation with preoperative gait velocity (r = − 0.25, p = 0.003, Table 2), whereas for postoperative gait velocity, this correlation was seen for T-tau (r = − 0.33, p < 0.001, Fig. 5A), P-tau_181_ (r = − 0.25, p = 0.005, Fig. 5B) and NfL (r = − 0.25, p = 0.003, Fig. 5C).

Regression modelling was utilized for dichotomized biomarker concentration variables with MMSE performance and gait velocity outcomes as dependent variables (Table 3). Low CSF P-tau_181_ (cut-off 27.5 ng/l, OR 3.4) and T-tau (cut-off 211 ng/l, OR 3.7) patients were more likely to have MMSE above 26 preoperatively (p < 0.05). Comparable results were seen for postoperative MMSE results: patients in the low CSF P-tau_181_ (cut-off 27.5 ng/l, OR 4.5), T-tau (213.5 ng/l, OR 8.1), NfL (1045 ng/l, OR 3.7), Aβ_38_ (1710 ng/l, OR 2.5) and Aβ_40_ (3714 ng/l, OR 2.2) groups were more likely to have MMSE scores above 26 (p < 0.05), respectively compared to the scores of patients in the high concentration groups. Favourable outcome of 20% increase in gait velocity was 2.8-, 2.7-, 0.4- and 0.4-times more likely for low CSF T-tau (cut-off 206 ng/l), NfL (1050 ng/l), Aβ_42_ (301 ng/l) and sAPPβ (239 ng/l) concentration patients compared to high (p < 0.05). The most significant multivariate model for evaluating preoperative MMSE included P-tau_181_, NfL, *APOE*, sex and age (Nagelkerke R^2^ = 0.18, 69.8% accuracy). In this model, low CSF P-tau_181_ patients were 3.4 times more likely to have MMSE above 26 (CI (1.3–8.7), p = 0.01). For postoperative MMSE, the combination of T-tau, NfL, Aβ_38_, age and sex best predicted the performance (Nagelkerke R^2^ = 0.32, 72.9% accuracy). The low CSF T-tau and NfL patients were 13.6 (CI 3.4–55.0) and 2.4 (1.0–5.8) times more likely to have MMSE above 26 (p < 0.05). The model with a more specific CSF T-tau cut-off (306 ng/l) predicted MMSE to be below 26 postoperatively by 96.7% specificity, 37.0% sensitivity and 63.9% overall accuracy. In this model, high T-tau patients had a 17.0-fold (CI 3.8–75.3, p < 0.001) greater risk of having MMSE below 26 postsurgery. Gait improvement over 20% postoperatively was best predicted by T-tau, sAPPβ, *APOE*, age and sex (Nagelkerke R^2^ = 0.22, 75.8% accuracy). The patients with low CSF T-tau and absence of *APOE* ε4-allele were 3.1 (1.0–9.4) and 3.9 (1.4–10.4) times more likely to improve their gait by 20% postsurgery (p < 0.05).

**Table 3:** Regression modelling of biomarkers predicting MMSE scores over 26 pre- and postoperatively and gait velocity improvement over 20% postoperatively

MMSE ≥ 26 preoperative							
Univariate	Odds	*p*-value	OR	C.I. (OR)	Predicted	R-squared	Model *p*-value
P-tau < 27.5	1.23	.001*	3.41	1.62–7.20			
T-tau < 211	1.31	.001*	3.69	1.66–8.24			
NfL < 1317	0.71	.069	2.02	0.95–4.33			
Aβ38 < 1692	0.74	.068	2.10	0.95–4.64			
Aβ40 < 4461	0.67	.111	1.95	0.86–4.43			
Aβ42 < 404	0.41	.270	1.51	0.73–3.13			
sAPPα < 241	0.59	.238	1.80	0.68–4.78			
sAPPβ < 317	− 0.37	.308	0.69	0.34–1.40			
MCP1 < 493	− 0.39	.281	0.68	0.33–1.38			
Multivariate: P-tau, NfL, Age, Sex, *APOE*					69.8	.183	.011*
P-tau < 27.5	1.22	.011*	3.38	1.32–8.70			
NfL < 1317	0.73	.165	2.07	0.74–5.78			
*APOE *ε*4* absent	0.26	.608	1.29	0.48–3.47			
Age	− 0.03	.348	0.97	0.92–1.04			
Sex (male)	0.15	.741	1.16	0.47–2.87			

MMSE ≥ 26 postoperative							
P-tau < 27.5	1.50	.000*	4.49	2.17–9.31			
T-tau < 213.5	2.09	.000*	8.05	3.58–18.11			
NfL < 1045	1.31	.000*	3.69	1.82–7.46			
Aβ38 < 1710	0.92	.016*	2.51	1.19–5.30			
Aβ40 < 3714	0.78	.024*	2.18	1.11–4.28			
Aβ42 < 407	0.30	.397	1.35	0.68–2.68			
sAPPα < 435	0.37	.288	1.44	0.74–2.82			
sAPPβ < 260	0.45	.194	1.56	0.80–3.07			
MCP1 < 515	0.42	.249	1.52	0.75–3.12			
Multivariate: T-tau, NfL, Aβ38, Age, Sex					72.9	.321	.000*
T-tau < 213.5	2.61	.000*	13.61	3.37–54.99			
NfL < 1045	0.88	.049*	2.40	1.01–5.75			
Aβ38 < 1710	-0.94	.172	0.39	0.10–1.50			
Age	0.03	.424	1.03	0.96–1.10			
Sex (male)	0.21	.623	1.23	0.54–2.82			

Gait improvement > 20%							
P-tau < 27.5	0.65	.118	1.91	0.85–4.27			
T-tau < 206	1.01	.016*	2.75	1.20–6.27			
NfL < 1050	1.00	.017*	2.73	1.20–6.22			
Aβ38 < 1255	− 0.64	.116	0.53	0.24–1.17			
Aβ40 < 3188	− 0.36	.382	0.70	0.31–1.57			
Aβ42 < 301	− 0.86	.034*	0.43	0.19–0.94			
sAPPα < 271	− 0.80	.099	0.45	0.17–1.16			
sAPPβ < 239	− 0.94	.024*	0.39	0.17–0.89			
MCP1 < 504	0.48	.242	1.61	0.73–3.56			
Multivariate: T-tau, sAPPβ, Age, Sex, *APOE*					75.8	.221	.008*
T-tau < 206	1.14	.043*	3.13	1.04–9.43			
*APOE *ε*4* absent	1.35	.008*	3.85	1.43–10.36			
sAPPβ < 239	− 1.01	.076	0.37	0.12–1.11			
Age	0.02	.654	1.02	0.95–1.09			
Sex (male)	0.25	.624	1.28	0.47–3.48			

Significant results are highlighted with asterisk (*). Biomarkers were transformed to dichotomous variables with ROC derived cut-offs for biomarker concentration in CSF (used cut-off presented as ng/l and after each biomarker). Odds and odds ratios are calculated for low concentration group predicting the MMSE score over 26 and gait velocity improvement over 20%. The model prediction accuracy is presented in predicted column. Explained variance is evaluated by Nagelkerke R^2^ and presented in R-squared column. Age and sex were used as covariates for analysis. The multivariate models are the best combination of variables to explain the variance and predict the MMSE performances and gait improvement. *APOE* dichotomized by presence of allele ε4. Results are presented for patients with no *APOE* allele ε4. OR: odds ratio; MMSE: mini mental state examination; Gait: gait velocity in m/s; T-tau: total tau protein; P-tau: phosphorylated at threonine 181 tau protein; NfL: neurofilament-light; Aβ38: Amyloid-β 38; Aβ40: Amyloid-β 40; Aβ42: Amyloid-β 42; sAPPα: soluble amyloid precursor protein α; sAPPβ: soluble amyloid precursor protein β; MCP1: monocyte chemoattractant protein 1; *APOE* ε4: apolipoprotein E allele ε4

In the follow-up from pre- to postsurgery, both cohorts improved in NPHGS (p = 0.025) and NPHS (p < 0.0001). In the subdomains of both NPHGS and NPHS, a significant change was seen in gait (p = 0.01 FIN, p < 0.0001 SWE), along with cognition (p < 0.0001). In the Swedish cohort, P-tau_181_ was the only biomarker to correlate with the NPHS outcome (r = − 0.25, p = 0.028). In the Finnish cohort, MCP1 (r = − 0.28, p = 0.030) correlated with postoperative outcome. Interestingly, MCP1 was found to negatively correlate with the incontinence outcome of NPHGS in the Finnish cohort (r = − 0.34, p = 0.009).

## Discussion

To our knowledge, this is the first study to report this broad preoperatively-collected CSF biomarker profile of iNPH patients in comparison with their MMSE scores and gait velocity. Our data showed that T-tau, P-tau_181_ and NfL are associated with cognitive and gait performance in iNPH patients. Furthermore, T-tau and NfL were able to predict postoperative MMSE results, and T-tau was able to predict an improvement of 20% or more in postoperative gait velocity.

The role of biomarkers in defining clinical symptoms has not been established previously with iNPH patients. Nonetheless, our findings verify previous results of the ability of preoperatively obtained lumbar CSF P-tau_181_ to predict postoperative MMSE values and thus support the ability of T-tau and P-tau_181_ to predict postoperative cognition [12–14]. Contrary to Nakajima et al. [13], who reported that P-tau_181_ correlated only to the postoperative MMSE, we found P-tau_181_ to correlate with both pre- and postoperative MMSE values. This difference may be due to the larger number of patients within our study. Additionally, we performed CSF P-tau_181_-derived group comparison (27.5 ng/l cut-off) against the pre- and postoperative MMSE values. The results were similar to those previously found [13], as the MMSE performance of patients in the groups differed significantly, with a higher P-tau_181_ group resulting in significantly worse postoperative MMSE score (Fig. 4). In logistic regression modelling, we found similar results using ROC curve-derived cut-offs (P-tau_181_ 27.5 ng/l, T-tau 213.5 ng/l), where the low CSF P-tau_181_ and T-tau groups had a significantly increased chance of MMSE above 26 postoperatively (Table 3). In previous studies [11, 13], T-tau and P-tau_181_ could predict gait outcome measured with NPHGS. Since the gait domain of NPHGS is derived mostly from gait velocity, we consider our results to partly support these earlier findings.

For amyloid precursor protein-derived biomarkers, we found no direct association with shunt response, corroborating the results of a previous study [25]. However, our postoperative MMSE values did not show a correlation with CSF Aβ_42,_ as previously reported [14]. Interestingly, in our data, the MMSE outcome was found to correlate positively with preoperative Aβ_42_ (Fig. 3C). Regarding the *APOE* genotype, we found a weak association with CSF Aβ_42,_ as expected. In regression modelling, the absence of the *APOE* ε4 allele predicted gait improvement of 20% postsurgery. The correlation seen for CSF MCP1 to outcome and incontinence in the Finnish cohort was interesting as well, evoking the question of whether underlying inflammatory mechanisms are frequently seen amongst Finnish iNPH patients. This finding was not seen in the Swedish cohort contesting this result to occur by coincidence.

The NfL concentrations showed similar associations with MMSE and gait velocity values as T-tau and P-tau_181_. In logistic regression modelling, patients with low CSF NfL were significantly more likely to have postoperative MMSE scores above 26 (Table 3). Furthermore, the correlations of NfL to T-tau and especially to P-tau_181_ (Table 2) were notably lower than those between T-tau and P-tau_181_. We consider this difference to derive from the different origins of NfL being a large calibre myelinated axon protein [26]. Thus, reinforcing the role of NfL as reflecting changes in neurodegeneration independently from T-tau and P-tau_181_. In previous studies, NfL has been stated to be elevated in several neurodegenerative diseases, including iNPH, when compared to healthy individuals, mirroring both acute and subacute changes in brain metabolism [26–28]. Consequently, we believe that our baseline concentrations of lumbar CSF NfL are able to reflect symptoms seen both pre- and postoperatively.

A possible explanation for the prognostic features of T-tau, P-tau_181_ and NfL reported is their ability to reflect acute and subacute neuropathologic processes [28, 29]. The natural course of iNPH is reported to be progressive and potentially lethal [3, 30]. The associations between increased T-tau, P-tau_181_ and NfL CSF concentrations and more severe symptomatology probably represent ongoing damage to the brain parenchyma. More precisely, the progressive neurodegeneration seen in iNPH might derive from reported impaired CSF circulation [2, 31]. With shunt surgery accomplished, disease progression is restricted, but the damage to the parenchyma occurring presurgery remains to some extent, leading to lower MMSE values and slower gait velocity.

The other, more frequently discussed hypothesis for the negative correlation of CSF tau proteins, especially P-tau_181_, with the MMSE and gait velocity observed postoperatively is potentially caused by other underlying neuropathological processes, such as AD. Low Aβ_42_ and elevated P-tau_181_ concentrations of CSF are characteristics of comorbid AD in iNPH [32]. This hypothesis is supported by the results presenting the high prevalence of AD comorbidity in iNPH patients [7]. In addition, the CSF P-tau_181_-derived group comparison (Fig. 4) visualizes the varying outcome of MMSE with higher P-tau_181_ concentrations obtained presurgery. When comparing the Aβ_42_ compositions between the P-tau_181_ groups, a consistently lower concentration of Aβ_42_ was seen in the high-P-tau_181_ group. On the other hand, the improvement of MMSE in the low-P-tau_181_ group might present the established hypothesis of pure iNPH pathophysiology with reversible cognitive impairment since the low CSF tau proteins are stated to be a characteristic domain for iNPH [9, 33]. Regarding these hypotheses and results obtained here, we consider the biomarkers of neurodegeneration to have prognostic features that emerge during the postoperative follow-up.

**Fig. 6:**
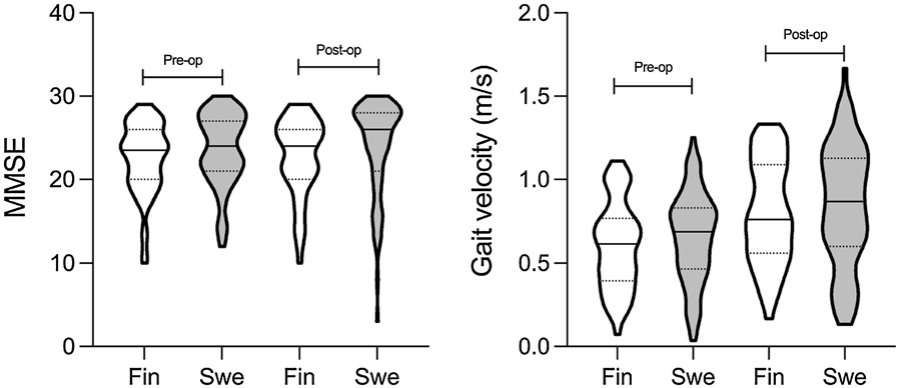
Pre- and Postoperative Mini Mental State Examination & Gait velocity distributions between cohorts. Pre- and post-operative MMSE results with gait velocity distributions of Finnish and Swedish cohorts are presented as violin plots. Violin plots including Kernel density plot and box plot combined. No significant differences were seen between the cohorts. MMSE: mini mental state examination; Pre-op: preoperative; Post-op: postoperative; Fin: Finnish cohort; Swe: Swedish cohort

**Fig. 7:**
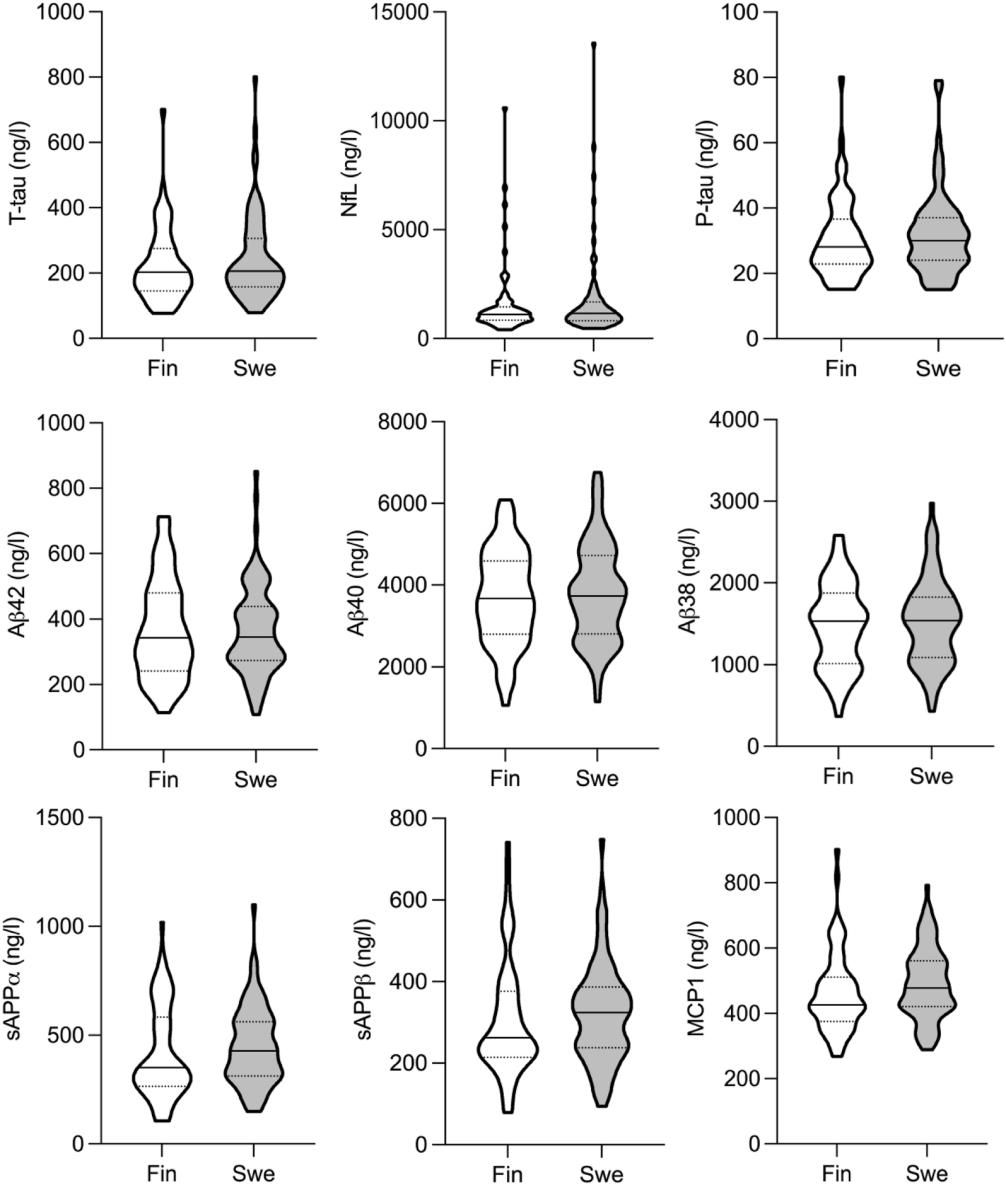
CSF biomarkers of neurodegeneration distributions in cohorts of Finnish and Swedish patients. Pre-operatively obtained CSF biomarker distributions presented as violin plot from Finnish and Swedish cohorts. Violin plots including Kernel density plot and box plot combined. No significant differences were seen between the groups. T-tau: total tau protein; P-tau: phosphorylated at threonine 181 tau protein; NfL: neurofilament-light; Aβ38: Amyloid-β 38; Aβ40: Amyloid-β 40; Aβ42: Amyloid-β 42; sAPPα: soluble amyloid precursor protein α; sAPPβ: soluble amyloid precursor protein β, MCP1: monocyte chemoattractant protein 1; Fin: Finnish cohort; Swe, Swedish cohort

Our second intent was to determine plausible differences between two separate cohorts of iNPH patients from different populations of Sweden and Finland. Our data showed that both cohorts were comparable in baseline characteristics and had equivalent shunt surgery outcomes despite the diverse diagnostic protocols (Table 1, Fig. 6). Moreover, the preoperatively obtained CSF biomarker concentrations and distributions were similar (Table 1, Fig. 7). These results add support to the notion that diagnostic guidelines and standardized clinical diagnostic procedures lead to the identification of patients with iNPH in a similar and reproducible way, which is of importance in the clinical setting but also for the generalization of results in research studies. Intriguing differences were seen when comparing the subdomains of NPHGS and NPHS from pre- to postsurgery. The Swedish cohort improved significantly in subdomains of gait and cognition, whereas significant improvement was only seen in gait for the Finnish cohort. This variety seen between different diagnostic tools might originate from the wider measurement range of the iNPH scale. Nevertheless, this comparison provides insight into how the positive shunt surgery outcome is mainly derived from gait and cognition improvements.

Our challenge in this study was the various measures for symptoms since the Kuopio University Hospital patients were graded using iNPHGS and the Sahlgrenska University Hospital patients were graded using the iNPH scale although the diagnostic tools of MMSE and gait velocity used in both centres were comparable. However, using the MMSE only most likely fails to consider all the cognitive dimensions seen with iNPH patients [34]. Furthermore, symptom validation using gait velocity can include confounding components since the measurement with metres per second does not take into account assistant devices used during the walk test. Nonetheless, we consider this not to notably influence our results, since the gait velocity outcomes were similar in both cohorts, and we had a relatively large study population as a whole. Another challenge we found, was not to be able to provide distinct biomarker thresholds below which MMSE improvements do not occur due the overlap of CSF biomarker concentrations between responders and non-responders. We also note that our follow-up times varied between the patients. Nonetheless, the variation seen between follow-up times was rather small.

We performed univariate and multivariate logistic regression modelling with ROC curve-derived biomarker cut-offs to validate the best predictive CSF biomarker combination for pre- and postoperative MMSE among patients with over 20% improvement in postoperative gait velocity (Table 3). To our surprise, P-tau_181_ was the best predictor of preoperative MMSE scores. NfL and T-tau combined with Aβ_38_ predicted postoperative MMSE results with the highest accuracy. The notable finding was also that a higher T-tau cut-off of 306 ng/l could predict at least mildly demented patients postoperatively by a specificity of 96.7%. In gait improvement, T-tau and *APOE* genotype were found to be the only significant variables in the multivariate model to have the ability to predict 20% postoperative gait improvement. These results highlight the usefulness of NfL- and tau-derived CSF biomarkers. Moreover, a better prognostic value is achieved by using the combination of CSF biomarkers that are reflecting different components of neurodegenerative processes. However, we cannot rule out shunt operations based on this study and further research is still needed for the clinical applications of these biomarkers.

## Conclusions

The patients in the study cohorts were comparable in baseline characteristics and showed equivalent shunt surgery outcomes. CSF biomarkers of neurodegeneration appeared to correlate with pre- and postoperative cognition, providing a window into neuropathological processes. CSF T-tau and P-tau_181_ appear to be the best predictors both for pre- and postoperative MMSE scores. In addition, preoperative T-tau may have potential for the prediction of gait velocity outcomes after shunt surgery.

## Data Availability

All data supporting the findings within this article will be shared anonymized by request of any qualified investigator.

## Abbreviations

iNPH: Idiopathic normal pressure hydrocephalus
AD: Alzheimer’s disease
CSF: Cerebrospinal fluid
DM2: Diabetes mellitus type 2
MMSE: Mini mental state examination
Pre-op: Preoperative
Post-op: Postoperative
*APOE*: Apolipoprotein E
NPHGS: Idiopathic normal pressure hydrocephalus grading scale (0–12)
NPHS: Idiopathic normal pressure hydrocephalus scale (0–100)
T-tau: Total tau protein
P-tau_181_: Phosphorylated at threonine 181 tau protein
NfL: Neurofilament-light
Aβ_38_: Amyloid-β 38
Aβ_40_: Amyloid-β 40
Aβ_42_: Amyloid-β 42
sAPPα: Soluble amyloid precursor protein α
sAPPβ: Soluble amyloid precursor protein β
MCP1: Monocyte chemoattractant protein 1
r: Pearson correlation coefficient
OR: Odds ratio
